# RETRACTED: Miranda et al. The Oral Microbiota: Implications in Mucosal Health and Systemic Disease—Crosstalk with Gut and Brain. *Cells* 2026, *15*, 82

**DOI:** 10.3390/cells15222072

**Published:** 2026-11-13

**Authors:** Vincenzo Miranda, Kamilia Laarej, Carlo Cavaliere

**Affiliations:** 1 Clinical Research Unit, Guna S.p.a., 20132 Milan, Italy; k.laarej@guna.it; 2 Department of Life Sciences, Health and Health Professions, Link Campus University, 00165 Rome, Italy; c.cavaliere@unilink.it

The journal retracts the review article “The oral microbiota: implications in mucosal health and systemic disease—crosstalk with Gut and Brain” [1] cited above.

Following publication, concerns were brought to the attention of the Editorial Office regarding the accuracy of the reference list presented in this article [1].

In accordance with the journal’s standard procedures, the Editorial Office and Editorial Board conducted an investigation, which identified numerous inaccuracies and inconsistencies within the reference list, including citations for which the referenced title, authorship information, and publication details did not correspond to the cited source. While the authors cooperated with the investigation and provided explanations regarding the identified issues, these explanations were deemed unsatisfactory by the Editorial Board. Given the extent of the concerns identified, the Editorial Board has lost confidence in the reliability of the findings and decided to retract this publication [1], as per MDPI’s retraction policy.

This retraction was approved by the Editor-in-Chief of the journal *Cells*.

The authors have been informed of this retraction and have indicated their disagreement with this decision.
